# Blood urea nitrogen/creatinine ratio in rhabdomyolysis

**DOI:** 10.4103/0971-4065.45295

**Published:** 2008-10

**Authors:** M. S. Walid

**Affiliations:** Medical Center of Central Georgia, 840 Pine Street, Suite 880, Macon, GA 31201, USA

**Keywords:** Acute renal failure, BUN, creatinine, potassium, rhabdomyolysis

## Abstract

Rhabdomyolysis, a potentially life-threatening syndrome, is not an uncommon condition with around 38,000 cases been reported in the USA in 2000. The risk of developing acute renal failure in the initial days of presentation can be as high as 40% and approximately 28–37% of patients require short-term hemodialysis. This report discusses a case of rhabdomyolysis after seizures in which blood urea nitrogen (BUN), creatinine, and BUN/creatinine levels were examined during the course of illness in the hospital. We found that the BUN/creatinine ratio is not a reliable indicator of renal function in rhabdomyolysis. Potassium levels can be a better marker for the early recognition of acute renal failure and an indication for prompt treatment with dialysis, which is crucial to prevent fatal complications.

## Introduction

Rhabdomyolysis is a potentially life-threatening syndrome. Interestingly, it is not an uncommon condition, with around 38,000 cases been reported in the USA in 2000.^1^ Most cases are caused by illicit drug/alcohol abuse (cocaine), muscular injury (trauma, crush injuries, heatstroke, shaking chills, marathon race, arterial occlusion), seizures, and myotoxic effects of prescribed drugs (statins). Rhabdomyolysis has been also reported in one case after severe hypokalemia (1.9 mEq/L).^2^ Renal dysfunction can complicate up to 30% of all rhabdomyolysis cases, and rhabdomyolysis is believed to be responsible for 5–25% of all cases of acute renal failure (ARF).^3^–^5^

In several cases, rhabdomyolysis has been reported to be a manifestation of primary HIV infection in which other potential causes of rhabdomyolysis were either present or not excluded. A recent report recommends considering HIV infection in every young patient who presents with acute rhabdomyolysis and fever, especially if he/she belongs to high-risk groups.^6^ Discussed here is a case of rhabdomyolysis after seizures in an HIV-negative patient that is unique for its abrupt onset and dramatic deterioration, but an overall satisfactory outcome. In this case, BUN and creatinine levels and BUN/creatinine ratios were examined during the course of illness in the hospital.

## Case Report

A 49 year-old African-American male was brought to the emergency room after having a seizure at home. The patient was confused and had chills and fever; he could not give any history. His girlfriend stated that the seizure occurred on the sofa and described it as mild. He smoked one and a half packs of cigarettes daily and was a rehabilitated ex-alcoholic. He looked thin and did not appear to be in any acute distress. His vital signs were the following: temperature 101.0°F, pulse 121/min, respiratory rate 20/min., blood pressure 109/73 mm Hg, and pulse oximeter 99% on room air. There was an abrasion across the tongue and some teeth were missing (old finding). Otherwise, the physical exam was within normal limits. Laboratory findings showed alcohol level < 10, white blood cells 6.2 ×10^3^/µL, hemoglobin 14.2 g/dL, hematocrit 41.5%, MCV 101.3 fL, platelets 153 × 10^3^/μL, neutrophils 75%, sodium 138 mEq/L, potassium 3.7 mEq/L, chloride 96 mEq/L, CO_2_ 15 mEq/L, glucose 142 mg/dL, BUN 10 mg/dL, creatinine 1.4 mg/dL, and calcium 8.7 mg/dL. CPK and myoglobin levels were highly elevated: 7215 μg/L and 6926 units/L, respectively; the phenytoin level was 3.5 μg/ml (subtherapeutic). Urinalysis showed pH 6, specific gravity 1.015, protein > 300 mg/24 h, positive for ketones, leukocyte esterase-negative, white blood cells 9 × 10^6^/L, red blood cells 78 × 10^6^/L, and hyaline casts 12/hpf. The urine drug screen was negative; HIV-1 antibody was absent. The chest X-ray showed no acute infiltrates and the CT scan of the brain did not reveal any abnormalities. Ultrasound showed echogenic kidneys bilaterally and cardiomyopathy with an ejection fraction of 25%. Kidney needle biopsy showed focal segmental glomerulosclerosis with collapsing features (glomerular obsolescence = 14%) and acute tubular necrosis. The above data were consistent with rhabdomyolysis. The patient was sedated with a titrated dose of Versed IV. Lumbar puncture was performed; the fluid looked clear with levels of glucose and protein of 89 mg/dL and 29 mg/dL respectively. He was loaded with Dilantin IV, had IV fluids, magnesium, and Ativan. Potassium levels rose to 5.7 mEq/L when the patient became totally oliguric with volume overload. The patient developed acute respiratory failure and required dialysis and ventilatory support. Blood, urine, and CSF cultures were negative but the patient was put on broad-spectrum antibiotic coverage. He was given multivitamins and sedated with benzodiazepine. He was discharged after 27 days in a satisfactory condition.

## Discussion

Rhabdomyolysis, which results in the release of large amounts of muscle cell contents into circulation, is a serious condition. Myoglobin, the 16.8 KDa hemoglobin-like protein, is freely filtered by the glomeruli and reaches the tubules, where it may cause obstruction, renal tubular necrosis, and most seriously, acute renal failure.

The risk of developing ARF in the initial days of presentation can be as high as 40%.^7^ Approximately 28–37% of patients require short-term hemodialysis.^5^

**Fig. 1:**
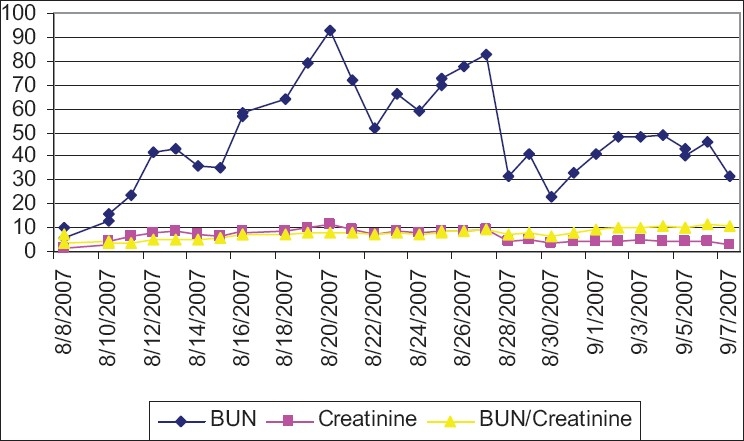
The levels of BUN, creatinine, and their ratio during the course of disease

The levels of BUN and creatinine, both markers of renal function, rise in cases of rhabdomyolysis complicated by ARF. Their ratio, as illustrated in our case, may not change significantly, or may even decline. The normal values for BUN are approximately 10–20, and 0.7–1.2 for creatinine. In our case, BUN levels rose to 93 and creatinine levels to 11.4 with the development of ARF. The normal ratio of BUN/creatinine is usually between 10 and 20 and in our case, it remained in the range of 3.5–11.2 [Fig. 1]. Thus, the BUN/creatinine ratio goes down in rhabdomyolysis because of the massive release of muscle creatinine into the blood. However, overriding acute kidney failure returns the ratio back to normal. These are two opposing factors that keep the BUN/creatinine level oscillating within normal and lower limits. Potassium level, on the other hand, reacts quickly and without oscillation to changes in renal function, therefore, it is a better predictor of impending ARF than BUN/creatinine. In addition, as its increase also brings up the risk of fatal arrhythmias, it is very crucial to monitor potassium level.^8^,^9^ *In vivo* experiments have shown that for each unit (mEq/L) increase in the initial potassium concentration, there is a 57% decrease in the chance of survival and that initial concentrations of BUN and serum creatinine are not prognostic.^10^ Keeping an eye on potassium levels along with continuous electrocardiographic monitoring is essential in all rhabdomyolysis patients.

## Conclusion

Given the high risk of ARF in rhabdomyolysis patients, it is important for physicians to monitor renal function appropriately using unfailing markers such as potassium level; the BUN/Creatinine ratio is not a reliable marker. Early recognition of ARF and prompt treatment with dialysis are crucial to prevent fatal complications.
